# Late initiation and low utilization of postnatal care services among women in the rural setting in Northwest Tanzania: a community-based study using a mixed method approach

**DOI:** 10.1186/s12913-021-06695-8

**Published:** 2021-07-02

**Authors:** Eveline T. Konje, Jennifer Hatfield, Reg Sauve, Susan Kuhn, Moke Magoma, Deborah Dewey

**Affiliations:** 1 Department of Biostatistics and Epidemiology, School of Public Health, Catholic University of Health and Allied Sciences, Mwanza, Tanzania; 2 Department of Community Health Sciences, Cumming School of Medicine, University of Calgary, Alberta Calgary, Canada; 3 Department of Paediatrics, University of Calgary, Alberta Calgary, Canada; 4 Engender Health Tanzania, Dar es Salaam, Tanzania; 5 Owerko Centre, Cumming School of Medicine, Alberta Children’s Hospital Research Institute, University of Calgary, Alberta Calgary, Canada; 6 Hotchkiss Brain Institute, Cumming School of Medicine, University of Calgary, Alberta Calgary, Canada

**Keywords:** Postnatal care, Maternal and child health, Timing on postnatal care, Low-and-middle-income countries

## Abstract

**Background:**

Maternal and newborn mortality is high immediately after childbirth and up to 42 days postnatally despite the availability of interventions. Postnatal care is crucial in preventing mortality and improving the health of women and newborns. This prospective cohort study investigated the initiation and utilization of postnatal care at health facilities and explored users’ and providers’ perspectives on utilization of postnatal care services.

**Methods:**

A sequential explanatory mixed method was used involving women who were followed from the 3rd trimester of pregnancy to 3–4 months postnatally in Northwest, Tanzania. From January to December 2018, a door-to-door survey was conducted 3–4 months postnatally among 1385 of these women. A convenience sample of women and community health workers participated in focus group discussions, and traditional birth attendants and nurses participated in key informant interviews to complement quantitative data. Data analyses were conducted using STATA version 13 and NVIVO version 12.

**Study findings:**

Approximately, one half of participants attended postnatal care within 42 days after delivery. Postnatal care seeking within 48 h after delivery was reported by 14.6 % of the participants. Women who attended antenatal care at least four times, delivered at health facilities or experienced delivery-related complications were more likely to seek postnatal care. Limited knowledge on the postnatal care services and obstetric complications after childbirth, and not being scheduled for postnatal care by health providers negatively influenced services uptake. Overwhelming workload and shortages of supplies were reported to hinder the provision of postnatal care services.

**Conclusions:**

Utilization of postnatal care services remains low in this setting as a result of a number of disparate and complex factors that influence women’s choices. Provision of effective postnatal care is hindered by lack of supplies, staffing, and inadequate infrastructure. To ensure accessibility and availability of quality services in this setting, both demand and supply sides factors need to be addressed.

**Supplementary Information:**

The online version contains supplementary material available at 10.1186/s12913-021-06695-8.

## Introduction

In the past two decades, significant reductions in maternal and neonatal mortality have been reported globally [1, 2]. However, disproportionately more women and newborns in low-and- middle-income countries remain at higher risk of preventable deaths than their counterparts in high-income countries [1–3]. Sub Saharan Africa accounts for 66 % of global maternal deaths due to pregnancy- and childbirth-related conditions such as severe bleeding, hypertensive disorder, infection, unsafe abortion, and birth complications [3, 4]. In 2017, Tanzania was among sixteen countries with the highest maternal mortality ratios, between 500 and 999 per 100,000 live births [3]. Globally, around 5.4 million under-five children died in 2017; 47 % of these deaths occurred during the neonatal period [2]. Conditions such as preterm complications, birth asphyxia, neonatal sepsis, or other neonatal conditions such as pneumonia, diarrhea, and tetanus account for approximately two-thirds of the neonatal deaths that occur in the first week of life [2, 5].

The postnatal period is a critical time for both women and their newborns [6]. It is defined as a period starting an hour after the third stage of labor and up to 42 days after delivery as per the World Health Organisation (WHO) guidelines that were adopted by the Tanzanian Ministry of Health, Community Development, Gender, Elderly and Children (MoHCDGEC) [6, 7]. The WHO and MoHCDGEC guidelines on postnatal care address timing, number and place of postnatal contacts, and content of postnatal care for all mothers and babies during the six weeks after birth and recommend that women and newborns be assessed for danger signs including maternal depression, provided with iron supplements, and counseled on hygiene, nutrition, exclusive breast feeding, newborn care, safe sex and family planning.

The postnatal period carries a higher risk of mortality that supports the importance of postnatal care for improving survival, reducing morbidity and improving quality of life [1, 2, 5, 8]. Timely and appropriate management of life-threatening conditions immediately after childbirth and postnatally have been shown to prevent mortality and morbidity [9–15]. Further, postnatal care encourages proper hygiene and nutrition, exclusive breastfeeding, breast care, skin and cord care, birth spacing, safe sex, promotion of healthy lifestyle and mother-baby interaction for both maternal and newborn well-being and survival [9, 16–20]. Lack of postnatal care has been associated with death and disability due to birth-related complications that have long term consequences for mothers and their children [9, 13, 14]. Delays in recognizing or identifying danger signs in either mothers or newborns can lead to untimely health seeking decisions and delays in reaching health facilities which in turn can impede the timely management of preventable causes of death [15].

A review by Victora et al., conducted in 2015 involving developing countries, reported that 58 % women and 28 % newborns in developing countries received postnatal care within 42 days after delivery. However, postnatal coverage differed significantly amongst countries [21]. According to the Tanzania Demographic and Health Survey (TDHS) for 2015-16, 44 % of women reported attending health facilities for postnatal care at least once within 42 days after delivery, 46 % of whom attended within two days after delivery [22]. However, there were significant disparities in postnatal care utilization by place of delivery and place of residence. Specifically, 60 % of the newborns delivered at health facilities received early postnatal check-ups (i.e., within 2 days after delivery) compared to only 10 % of newborns delivered at home. Further, a higher proportion of newborns from urban areas received early postnatal care (61 %) compared to newborns from rural settings (35 %) [22].

Studies have suggested that untimely initiation and low utilization of postnatal care is associated with limited awareness of postnatal care among women, perceived poor quality of care, and other socioeconomic factors [14, 15, 23–31]. Women perceived postnatal care to be for delivery-related complications, sick newborns, and vaccinations only [24, 26, 27, 31]. Thus, poor knowledge regarding the purpose of postnatal care and low uptake of postnatal care could partly contribute to the persisting high rates of maternal and neonatal mortality in Tanzania [22, 32].

To date, most of the studies that have examined the timing and utilization of postnatal care and factors associated with these in low-and-middle-income countries (LMIC) have been cross-sectional or used demographic and health survey data and are based on the responses of women who delivered 2–5 years previously. These studies may suffer from reporting and recall biases due to retrospective data collection [14, 23–26, 29–31, 33–35], which could be associated with either over or under reporting of postnatal care utilization [36]. To complement the existing literature, prospective studies using community-based surveys are required for an in-depth understanding of contextual factors underlying the sub-optimal uptake of services, including timing of utilization. We conducted a mixed method study to document factors influencing utilization, timing of initiation and provision of postnatal care services from the perspectives of both the users and the providers of the services in a rural district in Geita region Northwest Tanzania.

## Methods

### Study settings

This study was conducted in Geita district, which is located in Northwest Tanzania. It is one of six districts found in Geita region. Geita is among the regions with lowest level of utilization for maternal and child health services in the country [22]. In 2015/16 TDHS report, less than a half of pregnant women (48 %) delivered in health facilities and only 21 % of women received postnatal care within the first two days after delivery and only 22 % within 42 days after delivery [22]. During the study period (i.e. 2016–2018), the district had one hospital, five health centers, and 38 dispensaries. It was purposively selected due to its poor health services utilization indicators [22].

### Study design

A cohort of pregnant women from Geita district were recruited in their third trimester of pregnancy and followed at birth and 3–4 months postnatally (see S1 Fig.). Details on this cohort have been published elsewhere [37]. In brief, 1719 pregnant women were recruited in their 3rd trimester at the community level using a door-to-door survey. During the household survey, all pregnant women who were in third trimester were invited to participate. No sampling technique was applied in recruiting participants. The overall aim of this cohort study was to investigate the utilization of maternal and child health services, to examine pregnancy outcomes and birth-related complications, and to gain a deeper understanding of the sociocultural barriers associated with low utilization of these services from a community perspective.

The present study used a sequential explanatory mixed methods approach that included both quantitative (i.e., prospective cohort study) and qualitative (case study design) methods. This allowed for the qualitative findings to inform the quantitative results. Triangulation of these approaches can provide a deeper understanding of when women attended postnatal care services and potential factors that influence whether or not they attended. It also allowed us to determine which postnatal care services were provided to women who attended reproductive and child health (RCH) clinics at least once and whether the services received were consistent with the existing guidelines of the WHO and MoHCDGEC [6, 7].

### Quantitative data collection

In the present study, a home visit was conducted 3–4 months after birth. Only women who participated in both the initial household survey in their third trimester of pregnancy and the birth follow-up survey at 7 days postpartum were eligible to participate (*N* = 1385) (see S1 Fig.). Women who were found to have died prior to the 3–4 month postnatal follow-up visit were not included as we were unable to ascertain their utilization of health services (*N* = 11). Women who had delivered twins were not included in our birth follow-up survey at 7 days postpartum because of the higher risk to adverse outcomes associated with a multiple birth (*N* = 27). Home visits, which involved a door-to-door survey, took place between January and September 2018. For women who were not found at home during the door-to-door survey, call back visits were made up to three times. No incentives were provided to the women who participated in the study. The final sample of women who participated in the door-to-door surveys was 1164.

 Face-to-face interviews were conducted in participants’ homes using a structured pretested questionnaire. A trained research team comprised of the principal investigator, two nurses, and two interns conducted the door-to-door surveys. This team was not involved in the provision of postnatal care in the study area. Questions on initiation of postnatal care services after delivery, types of services women and newborns received at different visits, immunizations received, and history of morbidity that led to seeking care in the past 3–4 months preceding the survey were asked. To reduce reporting bias due to social desirability, for participants who reported attending postnatal care services at least once, information on care seeking behavior was extracted from the women’s reproductive and child health card number 4 (RCH-4 card) and the newborns’ clinic cards. If discrepancies were noted, data from the cards were accepted as the representative of the postnatal care received. Socio-demographic characteristics were extracted from the baseline survey data that was collected in the 3rd trimester of pregnancy and linked to postnatal data.

During the home visits, participants were counselled on the importance of postnatal care services and encouraged to seek postnatal care for themselves and their newborns. In rare cases, the research team escorted participants to the nearest facilities for curative services if a mother or a newborn baby was found to be ill. This visit to the health facility was not captured as postnatal care utilization. All other visits to health facilities for postnatal care services were considered and included as postnatal care visits.

No distinction was made between postpartum and postnatal care because < 1 % of women specifically reported receiving postpartum care. Initiation of postnatal care was the outcome of interest and was classified as follows: not attended, attended within 48 h, attended within 3–7 days, or attended between the second and sixth week after delivery. Early initiation for postnatal care was defined as a first contact with a health facility either for curative or preventive services within two days after delivery excluding services provided immediately after childbirth among women who delivered at health facilities. Since place of delivery has been linked to postnatal care utilization in previous researches [26, 30, 38], it was used as the main predictor of postnatal care service utilization and defined as health facility or elsewhere; the latter also referred to as home delivery.

### Power calculation and statistical analyses

In the TDHS Report 2015-16, only 10 % of home deliveries and 60 % of facility deliveries utilized postnatal care services within two days after delivery [22]. Based on a two proportions sample size estimation formula and using a 95 % confidence interval and 5 % tolerable error, we estimated that we would need < 300 participants to determine differences in utilization of postnatal care services by place of delivery. Therefore, our final sample of 1164 participants provided sufficient power.

Epidata version 3.1 was used for data entry and STATA version 13 was used for data analysis [39, 40]. Descriptive statistics are presented using percentages for categorical variables and means/medians for continuous variables. Throughout the data analysis, 5 % was considered as the level of statistical significance for determining an association between the variables. A multinomial logistic regression model (mlogit with rrr option) was used in bivariate and multivariate analyses. Due to the small number of participants who visited health facilities for postnatal care within 48 h after delivery, these participants and those who attended health facilities within one week after delivery were combined into one group. Estimated relative risk ratios with their 95 % confidence intervals were determined. The relative risk ratio > 1 suggests that women with a particular attribute were more likely to utilize postnatal care services within one week or within six weeks of delivery, compared to women who did not utilize postnatal care. A relative risk ratio < 1 suggests that women with a particular attribute are less likely to utilize postnatal care services within one week or within six weeks compared to women who did not use postnatal care services. The assumption of multicollinearity was checked with the Hausman-McFadden test.

### Qualitative data collection approach

A qualitative case study approach was used to gain a deeper understanding of activities, events, and issues influencing postnatal care seeking behavior and utilization, the timing of postnatal care and the types of services provided in these rural communities [41]. Various data sources were considered including women who delivered either at home or at health facilities, community health workers (CHWs), traditional birth attendants (TBAs), and nurses in charge of RCH units.

Women who delivered either at home or at a health facility represented the demand side. We involved CHWs, TBAs and nurses in charges of RCH units to represent the health services supply side. Data collection approaches included focus group discussions (FGDs) and key informant interviews (KII). A convenience sampling approach based on place of delivery was used to select women for the FDGs. Women were invited to the FDGs by the CHWs. Only CHWs who were available during the study period were invited to participate in FGDs. This data collection technique provided a dynamic environment in which participants could voice and discuss issues related to postnatal care freely [41]. Key informant interviews were conducted with nurses in charge of RCH units and TBAs who were considered knowledgeable on issues related to the provision and availability of postnatal care services [41]. Seventy-two participants took part in the FDGs and KIIs; 33 women, 28 CHWs, two TBAs, and nine nurses in charges of RCH units. Participants were not provided any incentives for participation. We conducted ten FGDs over a 4-month period (Sept – Dec 2017): five with women, and five with CHWs. Each FGD consisted of 5–7 participants and lasted for approximately 90 min. Eight FGDs were conducted at primary schools, village offices or in open spaces and two FGDs were conducted within health facility premises. Key informant interviews with nurses in change were conducted in their offices, whereas the interviews with TBAs’ were conducted in their homes. To ensure privacy and confidentiality non-participants were restricted from all FGDs and KIIs.

### Data collection procedure

We used a semi-structured questionnaire to guide the discussions and interviews. All FGDs and KIIs were conducted in Swahili, since all participants were fluent in spoken Swahili, which is the national language of Tanzania. The discussions and interviews were conducted by two females (PI & a nurse) and two males (a nurse & intern doctor) whose work experience in the health system facilitated their understanding of the participants’ comments. The FGDs and KIIs were recorded and field notes were written during data collection. The field notes were used during interpretation of the study findings to facilitate understanding of the group dynamics and the local context of postnatal care. We achieved saturation after 10 FDGs; therefore, no further FDGs were conducted.

### Data management and analysis

Two research assistants who were fluent in Swahili and English transcribed and translated the FDGs and KIIs. The principal investigator and a co-author reviewed the English versions of all transcripts for consistency. The principal investigator randomly crosschecked six transcriptions with the original recordings for verification purposes. NVIVO 12 was used to analyze the qualitative data after manual familiarization of the data [42]. Themes were derived from the data based on what participants said. Using thematic analysis, initial coding was used to develop the general description of the themes. The descriptions were used to guide derivation of the main themes, sub-themes, and sub sub-themes using an iterative approach [43]. This involved data familiarization, identification of the main themes, indexing, charting, mapping, and interpretation [43, 44]. A debriefing meeting was held with village leaders, health providers and non-participant CHWs to corroborate the themes that emerged as part of our assessment of the validity of the findings [45].

Findings from the quantitative and qualitative analyses were integrated using a narrative approach, referred to as a weaving [46]. This approach involves describing quantitative and qualitative findings together in a single report or within the same section to enhance interpretations of the results [46]. The results are presented using this approach. Quantitative findings are reported first followed by insights arising from qualitative findings. From the quantitative approach, we assessed the timing and uptake of postnatal care and the effect of place of birth on postnatal care utilization. The qualitative approach was used to gain a more detailed understanding of factors influencing postnatal care seeking behavior and utilization, the timing of postnatal care and the types of services provided in the study area from a community perspective.

## Results

A total of 1164 women participated in the study (see S1 Fig.). The women were 26 ± 6.63 years old, the majority (94.24 %) were married, and more than two-thirds (77.15 %) reported having at least primary education. More than half (53.18 %) of the women were either primigravida or grand multigravida. No significant differences were observed in demographic characteristics between the postpartum women followed up at 3–4 months and the original cohort of pregnant women recruited at the 3rd trimester (S1 Table). The majority of participants reported attending the recommended four antenatal care visits; however, less than a half (48.28 %) of the women delivered at a health facility. Pregnancy and labor-related complications were reported by 14.86 % of the women (see Table 1).

**Table 1:** General characteristics of 1164 postnatal women contacted during follow up

Characteristics		Number of women			
		**n (%)**	**Mean ± **^**a**^**SD**		
**Socio-demographic**					
Maternal age			26.04 ± 6.63		
Marital status	Single	67 (5.76)			
	Married	1097 (94.24)			
Education level	No schooling	266 (22.85)			
	Primary	807 (69.33)			
	Secondary and above	91 (7.82)			
**Pregnancy related**					
Gravidity	Primigravida	199 (17.10)			
	Multigravida(> 1but ≤ 5)	545 (46.82)			
	Grand Multigravida(> 5)	420 (36.08)			
Antenatal visits	Attended < 4 visits	494 (42.44)			
	Attended ≥ 4 visits	670 (57.56)			
Place of delivery	Home	602 (51.72)			
	Facility	565 (48.28)			
Any Pregnancy and labor related complication	Yes	173 (14.86)			
	No	991 (85.14)			

^a^*SD* Standard deviation

Findings on utilization and initiation of postnatal care, and the services that were reported to be received by women are presented in Table 2. Overall, early initiation for postnatal care services (i.e., attendance within the first two days after delivery) was limited to 14.57 % of the participants and less than a quarter of the women (23.59 %) attended within a week. Place of delivery was significantly associated with early initiation of postnatal care services. Only 6.03 % of home deliveries utilized postnatal care services within two days after delivery compared to 23.74 % of facility deliveries. There was also a significant difference between home and facility deliveries in terms of who attended postnatal care within the first week after delivery: 13.90 % vs. 33.99 % respectively. Overall, approximately half of the women (49.52 %; 95 % CI: 46.64 %, 52.41 %) attended postnatal care clinics within 42 days after delivery. However, significant differences were found between those who delivered at home and those who delivered at a health facility. Specifically, only 39.53 % of women who delivered at home attended postnatal care within 42 days after delivery compared to 60.25 % of women who delivered at health facilities. Few women (9.97 %) attended a health facility for postnatal care at least three times as per the WHO and MoHCDGEC guidelines. A significant difference was observed by place of delivery with a higher proportion of women who delivered at health facilities seeking postnatal care at least three times compared to women who delivered at home. Commonly reported postnatal care services received by women and their newborns were vaccination (Polio zero and BCG (Bacille Calmette-Guerin zero)), growth monitoring, and health education (specifically, counselling for hygiene, exclusive breast feeding, and family planning). Only a few women (*n* = 11, < 1 %), reported receiving curative services either for themselves or for their newborns.

**Table 2:** Usage of and first contact to postnatal care services by place of delivery (*N* = 1153)

Postnatal care utilization	Facility delivery	Home delivery	Overall	Chi^2^ *p*-value
	**n (%)**	**n (%)**	**n (%)**	
**Attended PNC at least once within 6weeks**				< 0.001
Yes	335 (60.25)	236 (39.53)	571 (49.52)	
No	221 (39.75)	361 (60.47)	582 (50.48)	
**First contact to PNC services**				< 0.001
Within two days	132 (23.74)	36 (6.03)	168 (14.57)	
3–7 days	57 (10.25)	47 (7.87)	104 (9.02)	
7–42 days	146 (26.26)	153 (25.63)	299 (25.93)	
Not attended	221 (39.75)	361 (60.47)	582 (50.48)	
**Total PNC visits**				< 0.001
None	221 (39.75)	361 (60.47)	582 (50.48)	
One	165 (29.68)	162 (27.14)	327 (28.36)	
Two	76 (13.67)	53 (8.88)	129 (11.19)	
Three and above	94 (16.91)	21 (3.52)	115 (9.97)	
**Received PNC services (*n* = 571)**				
Vaccination + growth monitoring			194 (33.98)	
Vaccination only			150 (26.27)	
Growth monitoring			217 (38.00)	
Health education (hygiene, FP,EBF)			77 (13.49)	
Other services			11 (1.93)	

*PNC Postnatal care, *FP* Family Planning, *EBF* Exclusive Breast Feeding

Low utilization of and different perspectives on the timing of postnatal care emerged as themes in the qualitative analyses. Key issues identified from the FDGs with the women that influenced initiation of postnatal care or first contact with a health facility were the health status of the woman or the baby or both. For those who delivered at home, the health condition of both the mother and her newborn was related to whether a woman sought postnatal care or not. The presence of birth-related complications was associated with immediately seeking postnatal care. If a newborn delivered at a health facility missed their vaccination, this precipitated a visit to a health facility within the first week after delivery. Hence, the timing for seeking postnatal care was associated with either adverse obstetric conditions experienced during labor and delivery by the women, the health status of the baby or the baby’s vaccination status. The women voiced these issues as follows:

*“We usually go to the health facility for vaccinations after three days if you gave birth at home, but if you gave birth at the health facility and the baby received vaccinations you will need to go back after a month for a second vaccination.“ Woman#3 (ward 1)*.

*“For a woman who gives birth at home and everything goes well with the delivery, she will not need to go to the facility till the baby is six weeks old.” Woman#6 (ward 2)*.

The CHWs and TBAs also noted that timing of postnatal care services for women and their newborn babies depended on the health condition of both the mother and her newborn as well as the baby’s vaccination status.

*”Women may come back to postnatal care at different times depending on the risk assessment during delivery. A woman who experienced complications during delivery or had a caesarean section will come back within seven days. Those with a normal delivery usually come back after six weeks. CHW-Female#5(ward 3)*.

*“The timing of postnatal care differs among women depending on their condition after delivery; if they don’t have any problems, they come back after six weeks.” CHW-Male#1 (ward 4)*.

*“Sometimes women who experienced problems or their babies are sick, may go back to the health facility immediately.” TBA#1 (ward 5)*.

In contrast, nurses in charge of RCH units had a different understanding regarding the timing of postnatal care. They noted that postnatal care should start immediately after delivery and include consecutive follow-up visits. Although nurses acknowledged the importance of three to four visits as stipulated in Tanzanian postnatal care guidelines adopted from the WHO, they thought that visiting at the sixth week may be more realistic due to current workload at the health facilities [6, 7]. They suggested that first contact soon after delivery and again after six weeks may be more realistic as coming more often would add to the workload at their respective health facilities. More than half of the women who reported utilizing postnatal care indicated that they came a week after delivery. This was acknowledged as typical postnatal care utilization by the nurses in charge of RCH units and was consistent with the information provided to women who delivered at health facilities at discharge.

*“Postnatal care should be provided soon after delivery, after 3 days, within 7–14 days and after six weeks. But we commonly provide postnatal care soon after delivery and women with their babies can come after six weeks for other services or when they have emergency health issues.”* (Nurse#2).

*“Postnatal care has 4 visits, but I don’t think that it applies here. We attend women mostly once but occasionally up to 3 visits depending on the condition of the mother and the* baby. *…” (Nurse#8)*.

*“…With the existing workload, we do multiple activities because of a limited number of staff. To minimize the load, we tell women to come back after six weeks unless they have health issues although this practice is not proper.” (*Nurse#9).

*“…we know the four visits but we can’t tell the women to come to all visits because it will add more work to us if they don’t have an emergency case.” (*Nurse#6).

*“… The number of postnatal visits need to be reduced as four visits are challenging for staff and women too…” (Nurse#8)*.

During postnatal period, women and newborns should be assessed for danger signs for severe bleeding, preeclampsia and eclampsia, infection, and other conditions that could potentially threaten their health. The participants in this study reported that provision of vaccinations and monitoring growth were only services commonly provided to newborns, and health education and counselling were provided to women. Women who had complications such as delivering by caesarian section were provided with relevant care but not all recommended components were provided. Women, TBA and CHWs understood and reported the limited components of the recommended care provided, which were indicative of a pervasive deviation from the policy recommendation by service providers.

*“There are no services for women after giving birth. We know services are there for children only. For us, we are only provided with information on hygiene, breastfeeding, and family planning” Woman#5 (ward 4)*.

*“The only postpartum care provided to women who come back after delivery either at seven days or six weeks is health education and counselling on breastfeeding, hygiene, and nutrition. No physical assessment is done to women unless a woman comes with a specific complaint.” CHW-Male#1(ward 6)*.

*“Services such as pressure measurement and folic/iron supplements are given only to pregnant women. Women will receive counselling on breastfeeding, nutrition, hygiene, and family planning after delivery”. CHW-Male#4 (ward 6)*.

In contrast, nurses in charge of RCH units mentioned additional postnatal services such as checking blood pressure, monitoring depression, and monitoring bleeding that should be provided to women and newborns but were not. The nurses in charge of RCH units understood the importance of the missed components of postnatal care services and attributed the pervasive inability to provide such components to lack of supplies, poor infrastructure, and shortage of staff.

*“The postnatal care services to be provided to women are many according to the visits. The most important ones are control bleeding after delivery, checking if the mother has a high fever, checking the blood pressure and vital signs, monitoring depression, and checking on lochia and general hygiene.”* (Nurse#6).

*“The postnatal care services provided to newborns include immediate monitoring of their breathing, managing emergency cases such as high/low temperature, cord bleeding, initiating early breast feeding, initiating immunizations after seven days and continuing with other immunizations after six weeks, and monitoring their growth after six weeks.”* (Nurse#4).

*“Challenges that we face in providing postnatal care include, unavailability of supporting tools, insufficient staffing, and poor infrastructure such as water and electricity.” (*Nurse#3).

*“…due to insufficient staffing, we are overworked by other duties. Women also have to wait in a queue for hours, and sometimes we don’t have immunizations and we refer them to other facilities.” (*Nurse#4).

*“…we are outnumbered by clients; sometimes the staff are busy with other out duties such as seminars…”* (Nurse#6).

The quantitative results showed that the presence of complications during labor and delivery, attending 4 or more antenatal (ANC) visits, and health facility delivery were significantly associated with timing of postnatal care (Table 3). Specifically, women who reported experiencing complications during labor and delivery were more likely to come for postnatal care within a week compared to those who had no complications. Women who delivered their babies at a health facility were more likely to contact health facility for postnatal care within the first week after delivery compared to those who delivered at home. In addition, early contact within the first week after delivery for a postnatal checkup was significantly higher among women with 4 or more ANC visits compared to those who had less than 4 ANC visits during pregnancy. Similarly, CHWs, TBAs, and nurses in charge of RCH units revealed that women with complications during labor and delivery, women with sick newborns, and those who delivered at the health facilities but whose newborns did not receive vaccinations at discharge were more likely to access postnatal care in a timely manner.

*“Women who had complications during delivery or those who had a caesarean section will receive services such as assessment of the wound and removal of the threads but others will not receive any services besides counselling for nutrition and breastfeeding” CHW-Female#2(ward 7)*:

*“Only when the labor was difficult and a baby has some problem, a woman and her baby may receive services after delivery. Most of the time, women will be counselled on how to breastfeed, use of family planning, hygiene, nutrition, and safe sex.” TBA#2 (ward 5)*.

**Table 3:** Factors associated with timing of postnatal services (bivariate & multivariate analyses)

Characteristics	No PNC	Within a week	>week to 6weeks	Within a week	>week to 6weeks
		**Bivariate analysis**		**Multivariate analysis**	
		RRR. (95 %CI)	RRR. (95 % CI)	RRR. (95 %CI)	RRR. (95 % CI)
**Maternal age**	Ref	0.99[0.97,1.01]	0.98[0.96,1.01]	-	-
**Being married**	Ref	1.09[0.59,2.05]	1.06[0.58,1.95]	-	-
**At least a primary level education**	Ref	1.48[1.13,1.95]	1.27[0.99,1.65]	1.22[0.90,1.63]	1.22[0.94,1.58]
**Gravida**					
**Multigravida**	Ref	1.19[0.78,1.79]	0.85[0.58,1.26]	-	-
**Grandgravida**	Ref	0.96[0.62,1.49]	0.78[0.52,1.16]	-	-
**Presence of complications**	Ref	1.68[1.70,2.44]	0.73[0.47,1.13]	1.28[0.87,1.92]	0.68[0.43,1.06]
**Less than 4 ANC visits**	Ref	0.55[0.41,0.73]	1.28[0.96,1.72]	0.57[0.42,0.78]	1.28[0.95,1.72]
**Home delivery**	Ref	0.27[0.20,0.36]	0.64[0.48,0.85]	0.29[0.21,0.40]	0.64[0.48,0.85]

*PNC* Postnatal care; *RRR* relative risk ratio; *CI* Confidence Interval; *ANC* Antenatal care

## Discussion

In this study, early initiation of postnatal care was an uncommon practice with less than 15 % of women accessing postnatal care within two days after delivery which is lower than the 21 % (Geita region) and 42 % (countrywide) as reported in the 2015-16 TDHS [22]. However, it is consistent with a study by Izudi and Amongin in Eastern Uganda that reported a 15.4 % postnatal care attendance within the first two days after delivery [33]. Approximately half of the women who participated in the present study reported attending health facilities for postnatal care within 42 days after childbirth compared to the 22 % uptake reported for Geita region and 46 % countrywide in the 2015-16 TDHS report [22]. The finding of the present study is also similar to that of 43.5 % reported in a community-based study conducted in southern Tanzania by Kanté et al. [24]. The discrepancies observed in existing research could be due to methodological differences among studies. Findings from prospective community-based surveys may yield different estimates compared to those from demographic and health surveys. This could be because in demographic and health surveys women are asked to recall events related to postnatal care utilization that occurred 2–5 years in the past. Due to social desirability or recall biases, the estimates of postnatal care uptake could result in overestimates or underestimates of actual utilization in these surveys [22, 25]. Further, the 2015-16 TDHS report disaggregated data to regional levels and not individual districts, which could partially account for the observed discrepancies.

Our findings reveal that women who delivered at health facilities, those who experienced birth-related complications, and those who completed at least four antenatal visits were more likely to utilize postnatal care within the first week after delivery. These findings are consistent with previous research, which has reported that women who delivered at health facilities, experienced complications or attended antenatal care were more likely to return for postnatal care within two or 42 days [25, 34, 35]. The knowledge imparted during antenatal care and at childbirth on the importance of postnatal care and recognizing danger signs after delivery could have a positive influence on the postnatal health seeking behavior of women who access these services. Further, women who delivered at health facilities could have been scheduled for postnatal care during discharge, which may have promoted attendance. On the other hand, women who delivered at home could fear that they will be reprimanded by health care providers, or may lack information on the benefits of postnatal care; hence, they may delay or not seek postnatal care [30, 38]. Limited knowledge of postpartum complications, not being scheduled to come for postnatal care and other health facility related factors such as suboptimal quality of care have been reported to negatively influence the care seeking behavior of women [27, 29, 31].

Previous studies have challenged the availability of effective postnatal care services in Tanzania [27, 47]. We observed that attending health facilities for maternal and child health services did not guarantee that women and their newborns received all of the recommended components of postnatal care. Based on the postnatal guidelines, women should be assessed for danger signs including vaginal bleeding, high fever, high blood pressure and mental health problems, provided with iron and folic acid supplements, and counselling on breastfeeding, nutrition, hygiene, family planning, and safe sex. However, in the present study, women reported that they were only provided with counselling. None of the women reported being assessed for danger sign, mental health problems or provided with iron and folic acid supplements. Health service providers also noted that counselling on breastfeeding, nutrition, hygiene and family planning were the only postnatal care services provided to women in this setting. For newborns, the postnatal care services provided were weight monitoring and vaccination while assessment of jaundice, cord infection, feeding well were missing. The health service providers acknowledged that they were not following the guidelines recommended by the MoHCDGEC for various reasons such as lack of necessary equipment, medicines and supplies, inadequate staffing levels and the overwhelming workload experienced by health providers were provided. Further, a number of service providers noted that due to the overwhelming service demands placed on them, they encouraged women not to initiate postnatal care until six weeks after delivery, which may be contributing to the pervasive non-prioritization by women of obtaining early postnatal care for themselves and their newborns in this setting.

It is notable that over 60 % of the women in our sample who delivered at home did not come for postnatal care within 42 days of delivery and that women who delivered at health facilities were more likely to come for postnatal care. These findings are consistent with a published study in Uganda that suggests the needed for promoting and strengthening health facility delivery to ensure women and their newborns are provided with early postnatal care prior to discharge from health facilities. Although, over half of the women who participated in the study did not deliver at a health care facility, all of the women attended antenatal care [48]. Therefore, promoting and educating women on the purpose and importance of postnatal care for themselves and their newborns during antenatal care visits could result in changes in health care seeking behavior and lead to increased postnatal care attendance. With increased access to cell phones, the use of mobile health technologies such as SMS and voice messaging in improving preventative maternal health care service utilization such as postnatal care needs further investigation [49]. Further, health care providers need to be educated on the importance of postnatal care in reducing maternal and child mortality and morbidity and promoting long term health and wellness. Such interventions, could result in health care providers being more enthusiastic promoters of postnatal care services to mothers and their newborns. It is essential to improve the quality of postnatal care provided to women and newborns in this setting by increasing the number of skilled health personnel and improving access to drugs, folate supplements, supplies, and equipment required to meet the increased demand. These increased resources would support health care providers in complying with the present recommendations of the Ministry of Health. It is also essential that health leaders at the district, regional and national levels are cognizant of the issues complicating delivery of postnatal care to women in rural setting and be involved in developing solutions that will assist health care providers in delivering services that are consistent with the recommended guidelines. Further, for all women, but particularly those who deliver at home, home visits in the first week after birth by community health workers who are educated in identify danger signs and delivering basic care for the mothers and newborns could significantly increase the recognition of postnatal problems and increase health-seeking behaviour among women, ultimately improving the quality of postnatal care [50]. This approach is consistent with the postnatal guidelines recommended by the WHO [6]. Finally, it is crucial that through education and policy change, the general understanding of the delivery of care provided during pregnancy, childbirth and the postpartum period evolves from one that sees of these different components separately, to one that perceives them as inter-related and contributing to the long term health of mothers and newborns.

### Strengths and limitations of the study

The main strengths of this study were the triangulation of the quantitative and qualitative findings and its prospective nature, which minimized recall bias among study respondents. An additional strength was its large sample size. This study also had some limitations. First, we only investigated postnatal care utilization in one rural district, whose context may differ with other districts in Tanzania and other low-and-middle-income countries. We also acknowledge the limited representation of influential people such as community leaders, district and regional reproductive and child coordinators, and members of council and regional health management teams who have major role in ensuring quality of services. Hence, future studies should include these stakeholders in order to tap from their knowledge and experience with ultimate goal of empowering them to systematically identify and address gaps within health system, particularly in relation to postnatal care. In addition, it is possible that participants in this study may have positively altered their health care seeking behavior as a result of close follow-up and home visits. However, the magnitude of this effect appears to be negligible as the prevalence of women attending postnatal care within 42 days after deliver in this cohort was found to be similar to that reported by other investigations in Tanzania [22, 24].

## Conclusions

Early initiation of postnatal care remains low in this setting with home delivery commonly practiced, which may act as a barrier to utilization of these services. Health care providers are not providing postnatal services as recommended in the MoHCDGEC guidelines. Further the services that are not provided such as folic acid supplementation, BP measurements, screening for maternal mental health are associated with maternal health outcomes. Hence, concerted efforts are required to address the challenges contributing to sub-optimal uptake and provision of postnatal care services in this setting. Due to shortages of skilled health care personnel in facilities, community health workers need to be empowered in the provision of postnatal care in women’s homes. On the job training in basic postnatal care and danger signs and supportive supervision by health care workers could assist in the provision of safe and appropriate postnatal care services in the community and reduce the significant demands placed on health care workers at health facilities. Addressing both health system factors such as lack of essential items for quality postnatal care, including inadequate service providers concurrent and demand side factors such as the relatively high level of home deliveries is essential to realize increased utilization and improved outcomes in relation to postnatal care.

## Supplementary Information

**Additional file 1:**

## Data Availability

The dataset and research materials from which conclusions are drawn are available upon request from the corresponding author.

## Abbreviations

ANC: Antenatal Care
CHW: Community health worker
CI: Confidence Interval
CUHAS: Catholic University of Health and Allied Sciences
EBF: Exclusive breast feeding
FGDs: Focused group discussions
FP: Family planning
KIIs: Key Informant Interviews
LMICs: Low and Middle Income Countries
MoHCDGEC: Ministry of Health, Community Development, Gender, Elderly and Children
PNC: Postnatal Care
RCH: Reproductive and Child Health
RRR: Relative risk ratio
SD: Standard deviation
TBAs: Traditional Birth Attendants
TDHS: Tanzania Demographic Health Survey
WHO: World Health Organisation
