# Supplementary material for: Late initiation and low utilization of postnatal care services among women in the rural setting in Northwest Tanzania: a community-based study using a mixed method approach
Source: BMC Health Serv Res. 2021 Jul 2;21:635. doi: 10.1186/s12913-021-06695-8 (PMC8252323; doi:10.1186/s12913-021-06695-8)
Supplement: Supplementary file 1 — Additional file 1: [file 12913_2021_6695_MOESM1_ESM.docx]

Late initiation and low utilization of postnatal care services among women in the rural setting in Northwest Tanzania: A community based study using a mixed method approach

Short title: Late initiation and low utilization of postnatal care in Tanzania

Eveline T. Konje^1,2*^, Jennifer Hatfield ^2^, Reg Sauve^2^, Susan Kuhn^3^, Moke Magoma ^4^, Deborah Dewey ^2,3,5, 6^

1. Department of Biostatistics and Epidemiology, School of Public Health, Catholic University of Health and Allied Sciences, Mwanza TANZANIA
2. Department of Community Health Sciences, Cumming School of Medicine, University of Calgary, Alberta CANADA
3. Department of Paediatrics, University of Calgary, Alberta CANADA
4. Engender Health Tanzania, Dar es Salaam TANZANIA
5. Owerko Centre, Alberta Children’s Hospital Research Institute, Cumming School of Medicine, University of Calgary, Alberta CANADA
6. Hotchkiss Brain Institute, Cumming School of Medicine, University of Calgary, Alberta CANADA

*Corresponding author

Email: [etkonje@ucalgary.ca](mailto:etkonje@ucalgary.ca) or [ekonje28@yahoo.com](mailto:ekonje28@yahoo.com) (EK)

**Supplementary Table and Figure**

**S1 Table: Comparing characteristics of 1719 pregnant women and 1164 postnatal women**

| **Characteristics** | | **Baseline at 3^rd^ trimester (pregnant women=1719)** | | **At 3-4 months follow up (postpartum women=1164)** | | | **p-value** |
| --- | --- | --- | --- | --- | --- | --- | --- |
|  |  | **Mean ± SD** | **n (%)** | **Mean ± SD** | **n (%)** |  |  |
| Maternal age |  | 25.73±6.60 |  | 26.04±6.63 |  | | 0.06 |
|  |  |  |  |  |  | |  |
| Marital status | Single |  | 91(5.29) |  | 67(5.76) | | 0.29 |
|  | Married |  | 1628(94.71) |  | 1097(94.24) | | 0.71 |
|  |  |  |  |  |  | |  |
| Education level | None |  | 406(23.61) |  | 266(22.85) | | 0.68 |
|  | Primary |  | 1179(68.59) |  | 807(69.33) | | 0.34 |
|  | Secondary + |  | 134(7.80) |  | 91(7.82) | | 0.49 |
|  |  |  |  |  |  | |  |
| Parity | Para0&4+ |  | 971(56.49) |  | 674(57.90) | | 0.23 |
|  | Para 1-4 |  | 748(43.51) |  | 490(42.10) | | 0.77 |
|  |  |  |  |  |  | |  |

**S1 Figure: The flow chart for recruitment and follow up of participants**

Door to door survey:

1805 were assessed for eligibility

1719 pregnant women in their 3^rd^ trimester recruited & followed up till birth

1429 mother infant pairs were visited within first week of birth

1385 of mother infant pairs were followed at 3-4 months postnatally

1164 of women were allocate

**Excluded (n=86)**

Not eligible (n=81)

Declined (n=2)

Other reasons (n=2)

**Excluded (n=290)**

Moved out of study area (n=71)

Couldn’t be located (n=33)

Other reasons (n=186)

**Excluded (n=44)**

Twins (n=27)

Missing information (n=17)

**Excluded (n=161)**

 Deaths (n=32)

Shifted (n=93)

Could not allocated (n=36)

**Excluded (n=11)**

Death (n=11)

1153 of women were included for analyses
